# Fatal Venous Gas Embolism During Endoscopic Retrograde Cholangiopancreatography After Simultaneous Deployment of 2 Self-Expandable Metallic Stents

**DOI:** 10.14309/crj.0000000000000873

**Published:** 2022-10-07

**Authors:** Justin Chuang, Rebecca Kuang, Ajit Ramadugu, Dipen Patel, Sachit Sharma, Kishan Shrestha, Jordan Burlen, Ali Nawras

**Affiliations:** 1 Department of Internal Medicine, University of Toledo, Toledo, OH; 2 Division of Gastroenterology and Hepatology, University of Toledo, Toledo, OH; 3 Division of Gastroenterology and Hepatology, Virginia Commonwealth University, Richmond, VA; 4 Nepal Medical College, Kathmandu, Nepal

## Abstract

Gas embolisms are a rare complication of endoscopic retrograde cholangiopancreatography (ERCP). While there have been multiple reports of ERCP-associated air embolisms, only 2 case reports using oral cholangioscopy and CO_2_ insufflation have been reported in the literature. We present a unique case of a fatal CO_2_ venous air embolism during ERCP without using cholangioscopy and with no intentional CO_2_ insufflation of the biliary tree.

## INTRODUCTION

Endoscopic retrograde cholangiopancreatography (ERCP) is a gastrointestinal procedure used in the treatment of biliary and pancreatic diseases.^1^ Although ERCP is a safe endoscopic procedure, it is associated with significant complications, most commonly post-ERCP pancreatitis.^2^ While extremely rare, complications such as perforation, pneumothorax, splenic injury, and air embolisms are difficult to identify and are associated with significant mortality.^3^ Gas embolisms are usually from air but can be from any gas (ie, carbon dioxide). In the current literature, there have been reported cases of both venous and systemic air embolism during ERCP. To the best of our knowledge, there have been at least 53 reported cases of ERCP-associated air embolisms, and at least 20 that were venous.^4,7^ Two of these were venous CO_2_ gas embolisms. We present the first reported case of CO_2_ emboli in ERCP without using cholangioscopy and with no intentional CO_2_ insufflation of the biliary tree.

## CASE REPORT

**Figure 1.**
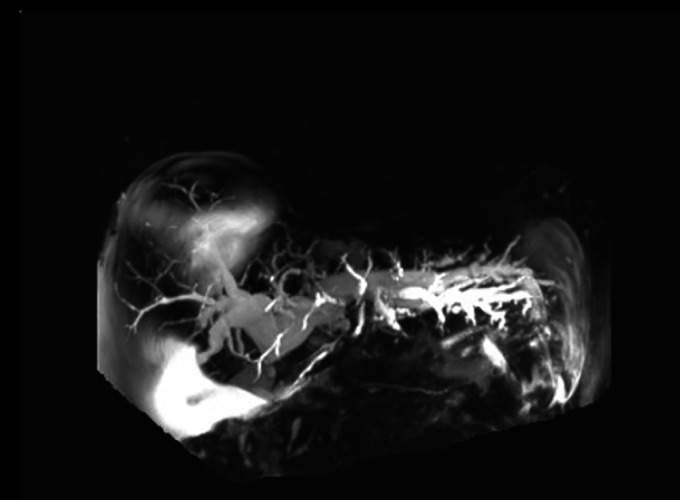
Magnetic resonance cholangiopancreatography image demonstrating a massive biliary dilation.

A 60-year-old man with a history of paroxysmal atrial fibrillation, alcohol-related liver cirrhosis, and ascites presented to ProMedica Toledo Hospital with a chief concern of jaundice and abdominal pain. On admission, initial laboratory test results showed a total bilirubin of 28 mg/dL and alkaline phosphatase of 493 U/L. Computed tomography of the abdomen/pelvis with contrast demonstrated significant intrahepatic biliary dilatation and multifocal nodularity throughout the omentum and peritoneum. Magnetic resonance cholangiopancreatography showed a stricture at the confluence of the intrahepatic ducts and cystic duct insertion as well as at progressive biliary dilatation compared with magnetic resonance cholangiopancreatography 2 months earlier (Figure 1).

**Figure 2.**
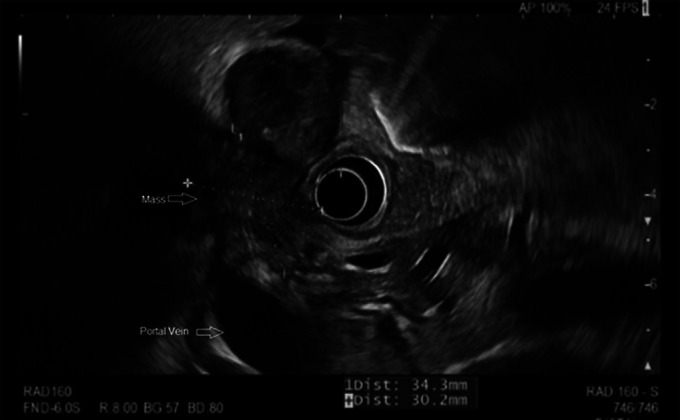
Endoscopic ultrasound image of the common hepatic/gallbladder mass.

Subsequent endoscopic ultrasound revealed a 34.3 mm × 30.0 mm hypoechoic, heterogeneous, and poorly demarcated common hepatic duct mass abutting/invading the portal vein (Figure 2). The mass was obstructing the common hepatic duct leading to diffuse dilation of the right and left intrahepatic biliary tree as well as compressing the duodenum and causing gastric outlet obstruction. A common hepatic duct stricture and duodenal stricture were also noted. The common hepatic duct stricture was biopsied and stented with 2 plastic biliary stents (85 mm × 15 mm) placed up to the left intrahepatic duct and the right intrahepatic duct after biliary sphincterotomy. The patient improved clinically with decreased jaundice and a downtrend of total bilirubin to 7.7 mg/dL from initial 28 mg/dL.

**Figure 3.**
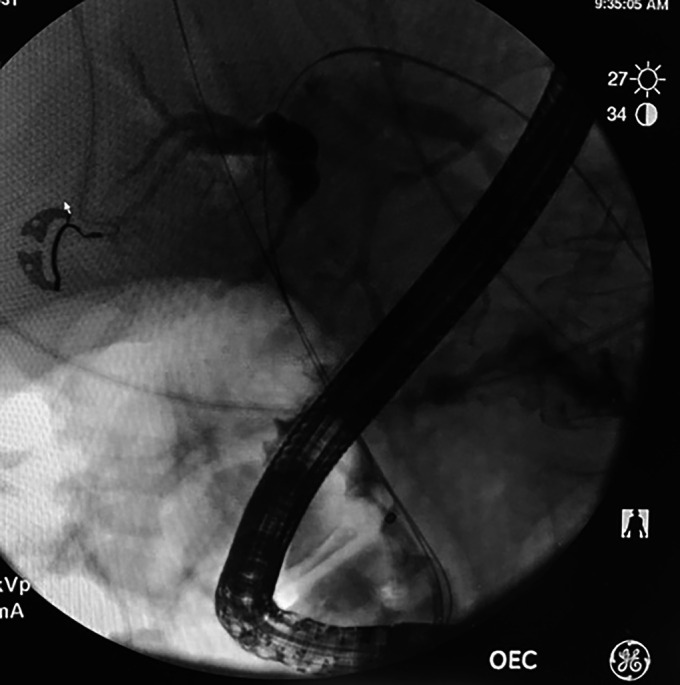
Cholangiogram of the second endoscopic retrograde cholangiopancreatography showing a biliary stricture.

**Figure 4.**
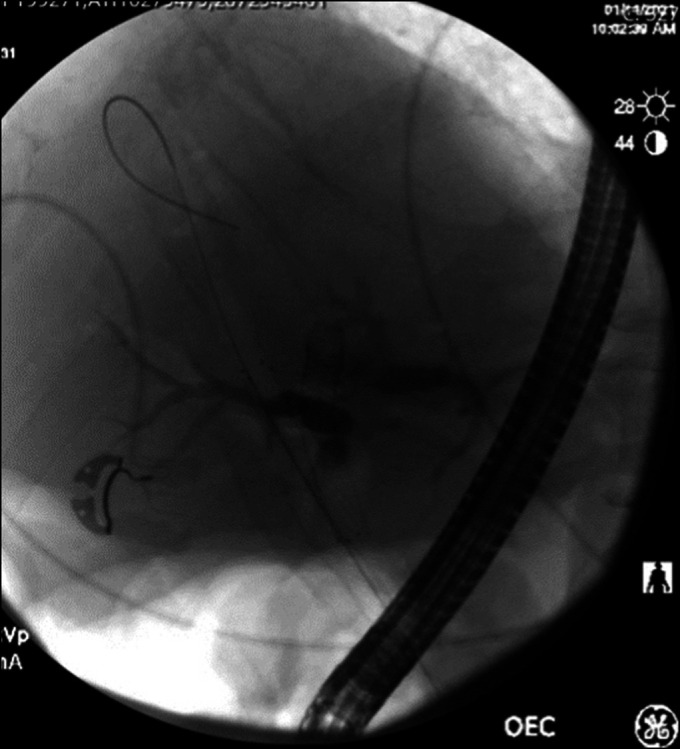
Two biliary endoscopic self-expandable uncovered metallic stents, 6 mm × 80 mm and 6 mm × 60 mm, were placed up to the left and right intrahepatic ducts, respectively.

Four days later, the patient underwent diagnostic laparoscopy with multiple biopsies obtained from the peritoneum, diaphragmatic surface, and omentum. Pathology was positive for adenocarcinoma of the gallbladder. A second ERCP was performed to place metallic common bile duct and duodenal stents 10 days after the initial ERCP. The previous 2 plastic biliary stents were identified and removed. Imaging from the second ERCP continued to show the biliary stricture (Figure 3). Two biliary endoscopic self-expandable uncovered metallic stents (6 mm × 80 mm and 6 mm × 60 mm) were placed up to the left and right intrahepatic ducts, respectively (Figure 4). Immediately after deploying the stents, the patient became hypotensive, bradycardic, and hypoxic. Cardiac resuscitation failed to achieve return of spontaneous circulation, and the patient unfortunately died. Transesophageal echocardiogram was performed during the code and revealed a massive gas embolism in the heart. All ERCPs were performed using carbon dioxide insufflation.

## DISCUSSION

While rare, gas embolisms are a potential complication with any endoscopic procedure and can be either venous or arterial. Several proposed mechanisms of venous gas entry during an ERCP include (i) direct high-pressure insufflation into the biliary system; (ii) leakage of air into the portal or hepatic venous system from the bile duct wall due to mechanical irritation from stones, endoscopy, contrast, or removal of bile duct stents; (iii) air entry into preexistent biliovenous fistula or biliovenous shunts; and (iv) air entry into adjacent veins from inflammation of the biliary/hepatic mucosa and muscular wall and communication through sphincterotomy to the portal vein.^4^ Risk factors of ERCP-associated venous gas emboli include previous hepatobiliary surgery, inflammation of the biliary tree or adjacent veins, altered papillary anatomy, history of previous stents, and others.^6^ A portal venous shunt could exist in a patient with cirrhosis complicated by hepatopulmonary syndrome. Our patient had no findings of this and had appropriate oxygen saturation on room air before the procedure.

This patient had multiple risk factors for mucosal compromise. These included metastatic adenocarcinoma of the gallbladder with a common hepatic duct mass that was invading the portal vein as well as a history of exploratory laparoscopy and ERCP with bilateral plastic hepatic duct stents and sphincterotomy in the preceding 10 days. He had both compromised anatomy and likely severe inflammation of the biliary tree because of his active gallbladder adenocarcinoma and recent sphincterotomy/stent placement. One other factor may include procedure time. Our case lasted for approximately 3 hours. Further research is required to investigate the relationship between procedure time and CO_2_ gas embolization because no current literature provides this correlation.

An explanation for our patient's acute decompensation at the time of simultaneous stent deployment may be due to an already compromised mucosa along with multiple risk factors, as mentioned before. This likely led to a perforation somewhere along the biliary tree and/or adjacent veins. Given that the adenocarcinoma was exerting a compressive effect after removal of the initial stents, the deployment of the new metallic stents opened the lumen so that gas was able to enter rapidly and travel through the portal venous system to the heart. Interestingly, CO_2_ is not normally a cause of venous embolism because intravascular CO_2_ is more easily absorbed than air.^7^ In fact, using CO_2_ insufflation has been shown to have a protective effect against gas embolism.^8^ Given that CO_2_ insufflation was used during ERCP for our patient, it is extremely likely that the combination of multiple risk factors and sudden patency of the hepatic ducts led to a fatal venous CO_2_ embolism. Although CO_2_ insufflation normally reduces the risk of gas embolism during ERCP, our report presents a unique case of a fatal CO_2_ venous air embolism.

## DISCLOSURES

Author contributions: J. Chuang and R. Kuang: study design, data acquisition and interpretation, and manuscript drafting. A. Ramadugu, D. Patel, S. Sharma, K. Shrestha, and J. Burlen: data acquisition and interpretation; manuscript drafting. A. Nawras: study supervision, critical revision for intellectual content. J. Chuang is the article guarantor.

Financial disclosure: None to report.

Informed consent could not be obtained from the family of the deceased. All identifying information has been removed from this case report to protect patient privacy.
